# The Use of Conditioning Open-Label Placebo in Opioid Dose Reduction: A Case Report and Literature Review

**DOI:** 10.3389/fpain.2021.697475

**Published:** 2021-07-12

**Authors:** Maria A. Estudillo-Guerra, Ines Mesia-Toledo, Jeffrey C. Schneider, Leon Morales-Quezada

**Affiliations:** Department of Physical Medicine and Rehabilitation, Harvard Medical School, Spaulding Research Institute, Spaulding Rehabilitation Hospital, Boston, MA, United States

**Keywords:** opioids, conditioning, pain, pharmaco-behavioral, open-label placebo

## Abstract

**Introduction:** Adequate pain management for inpatients in rehabilitation units is essential for achieving therapeutic goals. Opioid treatments are commonly prescribed, but these are associated with numerous adverse effects, including the risk of addiction and decreased quality of life. Conditioning an open-label placebo is a promising approach to extend the analgesic effect of the opioid while reducing its overall dosage.

**Objectives:** To describe a patient's experience in using conditioning open-label placebo (COLP) as a pharmaco-behavioral intervention to decrease opioid intake and its side effects after inpatient rehabilitation discharge, and to perform a literature review about the use of open-label placebo in pain.

**Methods:** This case study has been extracted from a clinical trial initiated in 2018. A 61-year-old male was recruited at a tertiary rehabilitation hospital after suffering a traumatic sport-related injury and orthopedic surgery. Pain management included prescription of non-steroidal anti-inflammatory drugs (NSAIDs) and short-acting oxycodone. After trial participation, the patient requested off-label COLP treatment to help him decrease outpatient opioid utilization.

**Results:** After COLP treatment, the patient could discontinue oxycodone intake (a reduction from 15 morphine equivalents/day) after rehabilitation discharge. Moreover, opioid side effects decreased from 46 to 9 points on the numerical opioid side-effects scale. A literature review identified five clinical trials using “honest” open-label placebo (OLP) or COLP as an experimental intervention for pain control. From these studies, two were in the area of chronic lower back pain, one in post spine surgery, one in irritable bowel syndrome, and another in spinal cord injury and polytrauma. Four studies reported positive outcomes related to pain control, while one study showed no significant differences in pain management between treatment-as-usual and the COLP group.

**Conclusion:** The case report illustrates how a pharmaco-behavioral intervention can facilitate downward opioid titration safely after inpatient rehabilitation. It initiates a discussion about new approaches for opioid management using conditioning and the patient's expectation of pain relief.

## Introduction

Pain management is one of the biggest challenges in rehabilitation medicine. Standard pharmacological treatment has a wide range of undesirable side effects. Despite the recommendation of avoiding opioids to control acute pain, and to prefer multimodal analgesia alternatives, opioids are often used as adjunctive therapy at the forefront of treating mild to severe pain (1].

Overreliance on opioids increases the length of stay and hospital costs while decreasing patient satisfaction. The risk of opioids usage is the highest of all common analgesic categories, the list is long and serious, including respiratory depression (the most serious immediate and short-term risk), dizziness, nausea, vomiting, ileus, constipation, sedation, delirium, hallucinations, falls, hypertension, aspiration pneumonia, delayed gastric emptying, sexual dysfunction, sleep disturbance, opioid-induced hyperalgesia, risk of addiction, cognitive impairment, and mood alterations (2–5).

Greater opioid prescribing contributes to increasing availability for abuse and overdose (6). Patients previously naïve to narcotics who receive opioids during hospitalization and after discharge are at an increased risk of becoming chronic opioid users (7). In comprehensive pain rehabilitation programs there is lack of evidence of long-term improvements in pain and functioning attributable to opioid therapy, as opioid-induced hyperalgesia and opioid tolerance can exacerbate pain, as well as the impact on functioning, mood, and pain catastrophizing (8).

The risk of addiction and chronic pain has played a central role in the current national epidemic of drug abuse. Therefore, novel interventions aimed at preventing addiction among inpatients undergoing intensive rehabilitation and receiving opioids for pain, are needed.

Classical conditioning has been used to induce analgesia through learned responses without the need for drugs (9), while placebo administration can also lead to pain relief. Therefore, the conditioning of placebo analgesia can be considered a promising approach to prevent opioid addiction in patients receiving opioid treatment.

Placebo analgesia occurs when a substance known to be non-analgesic produces an analgesic response) (10). The placebo effect is a widespread phenomenon in medicine. It manifests through various mechanisms which include: (1) cognitive factors like the expectation of pain relief which triggers the release of endogenous opioids; (2) classical conditioning mechanisms where repeated associations between an active agent and inert substance promote conditioned responses; and (3) factors related to the therapeutic encounter, such as empathy, enhanced communication, and a comfortable healing environment (11). Also, Accumulating data, suggested that placebo analgesia, appears in response to the individual expectations and subsequent conditioning (10, 12–15). The effect of expectation and outcome associations on placebo is mediated by endogenous opioid release and m-opioid receptor system activation in the dorsal anterior cingulate cortex (dACC) (16).

As observed in neuroimaging studies the placebo effects on pain, suggests the activation of prefrontal areas including (dlPFC), rostral anterior cingulate cortex (rACC), orbitofrontal cortex (OFC), and ventromedial prefrontal cortex (vmPFC), influence the pain by activating endogenous opioids pain regulatory mechanism pathways in the brainstem, in the periaqueductal gray (PAG) (17–24).

Unfortunately, the use of placebos in clinical practice has typically presented an ethical challenge because of their association with deception and concealment. On the other hand, open-label placebo (OLP), also known as an honest placebo, which consists of providing patients with a non-active substance without deception or concealment (25) while explaining the purpose and possible benefits, has already been demonstrated to provide analgesic effects in patients suffering from pain (24, 26–29).

The neurobiology of pain processing includes the sensory-discriminative aspects of pain, which are regulated by direct projections from spinal nociceptive neurons and the primary somatosensory cortex, posterior insular cortex, and thalamus (30). These nociceptive inputs are modulated and then transmitted to cortical and subcortical structures, such as the primary somatosensory cortex and posterior insular cortex, which are responsible to encode the intensity of painful stimuli (31). The brain regions associated with the affective dimension of pain processing include the secondary somatosensory cortex and anterior insular cortex (32), while cognitive modulation of the pain experience is thought to be driven largely by regions within the prefrontal cortex, placebo response is associated with the activation of this network (33).

The neural substrates of placebos involve the activation of diverse neural centers such as the rostral anterior cingulate, orbitofrontal and dorsolateral prefrontal cortex, anterior and posterior insula, nucleus accumbens, amygdala, thalamus, hypothalamus, and periaqueductal gray area (16, 34). Some of these regions overlap with those involved in mood regulation and motivated behavior (34), including those associated with pain and endogenous opioids release that mediates placebo analgesia (35). The endogenous opioid system plays an essential role in placebo analgesia and studies have confirmed that placebo analgesia can be blocked by the opioid antagonist naloxone (18). Conditioning-open label placebo (COLP) is an innovative pharmaco-behavioral approach, that has been previously used as a “dose-extender” on children with ADHD (36). In an open-label prospective crossover trial, placebos were paired with stimulant medication to elicit a placebo response in children with ADHD that allow them to be effective treated at 50% of their optimal stimulant dose. In this clinical case, we intended to follow the same principle of extending the analgesic effects of an opioid drug while decreasing its total dosage. Based on the classical conditioning and learning principles, this paradigm associates the active drug (e.g., oxycodone) with a neutral stimulus (placebo), so the analgesic effect is bound to the intervention. The placebo then becomes a conditioned stimulus triggering a conditioned response (placebo-driven analgesia). The patient's awareness of the placebo's inert nature enables caregivers to bypass any ethical issues related to deception or concealment.

The proposed model is a reinforced conditioning placebo paradigm that also introduces a sensory stimulus in the form of smell. Thus, the active intervention had the following assembly: opioid conditioning with a placebo pill plus an odorous stimulus as a booster of the learning experience.

## Case Description

We first contacted the patient when he participated in our COLP clinical trial [“Identifier: NCT03906721, Reduction of Opioid Dose Using Conditioning & Open-Label Placebo (COLP)”]. This trial explores the feasibility of a pharmaco-behavioral intervention for the reduction of opioids consumption in hospitalized patients. The patient was recruited in 2019, provided written informed consent, and was randomized to receive either COLP or treatment-as-usual (TAU) which was considered the control group. The randomization was performed using the order of entrance to the study and a previous randomization list generated by a computer using blocks of four, to minimize the risk of imbalanced groups.

After study participation, it was disclosed to the patient he was randomized to the TAU arm of the trial, the patient then asked if we would consider him for an off-label intervention after discharge. The patient was eager to try the open-label placebo intervention, as he was intrigued by the possible outcomes and aware of the side effects and risks associated with opioid intake.

**Figure 1:**
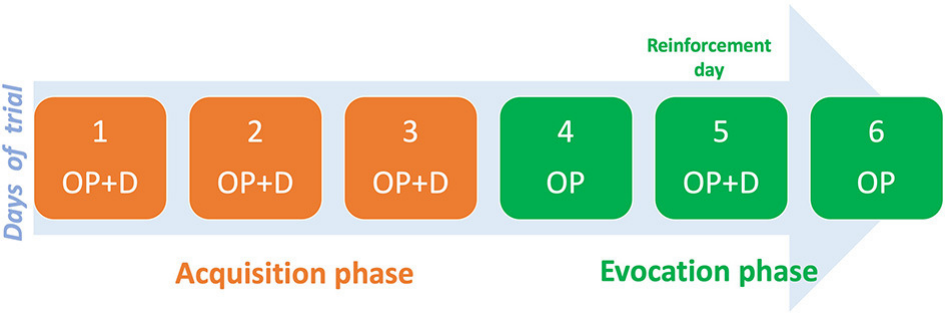
Open label placebo intervention. OP, open-placebo; D, opioid drug.

The COLP intervention entailed two phases: acquisition, and evocation. The acquisition phase consisted of administering short-acting oxycodone as needed (PRN) paired with the open-label placebo (inert capsule), and an odorous stimulus (smelling cardamom oil) for three consecutive days. This was followed by the evocation phase where, for two alternate days, the patient received only the placebo and the odorous stimuli in the same PRN scheme. Between the alternated days, the patient took both the placebo and oxycodone to reinforce the conditioning (Figure 1).

The patient approved his data to be used for this report. Pain intensity was measured using the standard VAS 11-point-scale on average per day, opioid dose consumption was registered using morphine equivalents and opioids intake side effects were evaluated using the Numerical opioids side effects (NOSE), a simple, rapid, self-administered instrument, that rates gastrointestinal issues, fatigue, itching, sexual function, among other side effects (37). All data were collected by a phone call daily until the off-label treatment.

## Diagnostic Assessment, Details Of The Therapeutic Intervention, Follow-Up, And Outcomes, As Specified In The Care Guidelines

### History of Present Illness

A 60-year-old white male (pseudonym “Dineen”) with relevant history of left knee osteoarthritis since 2017 and right knee patellar tendinitis since 2018 and no other relevant medical history, was admitted to the emergency department in 2019 after falling while running a marathon. He suffered a right intertrochanteric hip fracture (closed, minimally displaced). Soon after, an open surgical reduction with internal fixation was performed. After surgery, the patient was transferred to a tertiary rehabilitation hospital for intensive rehabilitation. During hospitalization, Dineen reported mild pain in his right hip. However, his main complaint was localized pain in the right knee, with an average intensity of 7/10 on the visual analog scale for pain (VAS). The patient described achy right hip, leg, knee pain starting post-operatively that worsen with the activity of movement and weight baring, repositioning for comfort and ice packs were applied.

During the hospitalization a right knee MRI was performed, where they reported absent anterior cruciate ligament (ACL), and laxity of the posterior cruciate ligament (PCL), consistent with remote rupture, and muscular strain of the biceps femoris. No joint effusion or synovitis, nor fracture, edema, osteonecrosis, or focal marrow replacing lesion was reported.

Inpatient pain management consisted of 3,250 mg/day of acetaminophen, 400 mg/day of ibuprofen, 2 mg/day of tizanidine, and 5–10 mg/3 hr of oxycodone PRN, with an average consumption of 35 mg/day (Table 1). After discharge, Dineen received 400 mg/day of ibuprofen, 5–10 mg/3 h of oxycodone PRN and COLP as an off-label intervention for opioid management. The treating physician and our research team supervised treatment compliance on daily basis. We followed up with the participant by phone, calling him every day and at the same time for consistency. The pain was reported as a daily mean value—average pain experienced during each day considering the pain fluctuation.

**Table 1:** Pain management over hospitalization, study participation and off label placebo intervention periods.

**Time**	**Days**	**Acetaminophen (mg dose)**	**Ibuprofen (mg dose)**	**Tizanidine (mg dose)**	**Oxycodone (morphine equivalent Dose)**	**VAS**
In patient	1	3,250			7.5	7
	2	3,250			15	6
	3	3,250			45	7
	4	3,250			75	6
	5	2,300			75	7
COLP study	6	3,000			45	5
	7	3,000			45	5
	8	3,000			15	6
	9	3,000			15	4
	10	3,000			15	5
	11	3,000		4	15	6
In patient	12	3,000	400	4	15	5
	13	3,000		4	15	5
	14	3,000	400	4	15	6
	15	1,000		4	7.5	6
Outpatient off label treatment	16		400		7.5	5
	17				7.5	5
	18		400		7.5	7
	19				0	7
	20				7.5	7
	21				0	5

*VAS, visual analog scale; COLP, Conditioning open label*.

**Figure 2:**
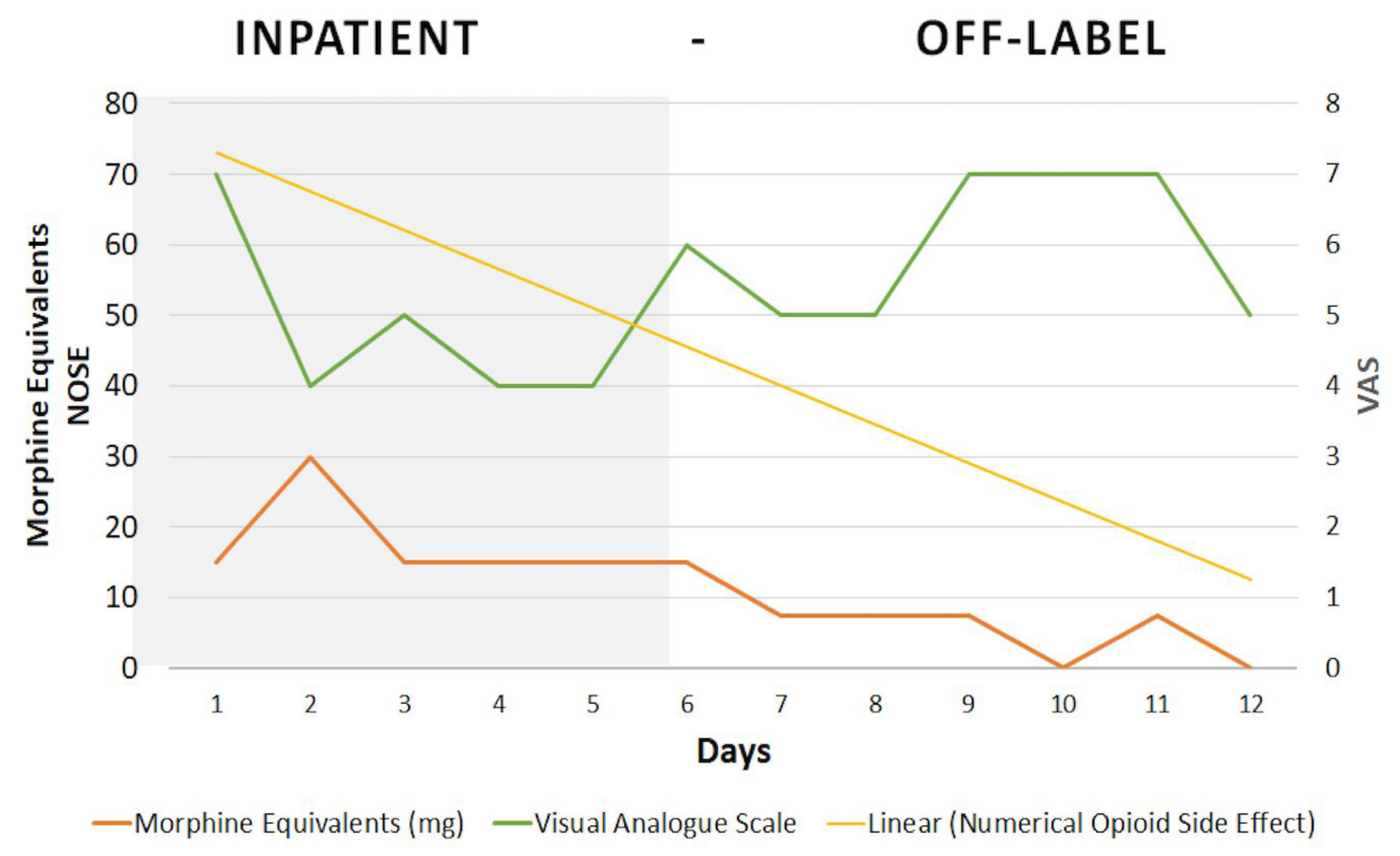
Results. NOSE, numerical opioid side effect; VAS, visual analog scale.

During the first 3 days after discharge (Figure 2), Dineen received an open-label placebo together with oxycodone (acquisition phase) and reported moderate pain (VAS = 5). This was followed by the placebo-only period (evocation phase) (days 4 to 6) and a two-point increase in pain was reported (VAS = 7). On the reinforcement day, severe pain (VAS = 7) was reported. During the COLP treatment period, opioid side effects showed a steady decrease on the numerical opioid side effects scale (NOSE), from 46 to 9 points (80% decrease), as the patient had discontinued use of as-needed oxycodone.

## Discussion

This case illustrates how a pharmaco-behavioral intervention can be safely used to condition opioid analgesic effects while decreasing total dose consumption and the associated side effects, within a relatively short period of 7 days. For the duration of the TAU period, Dineen kept his average opioid intake without changes at 15 mg/day. After starting the off-label placebo intervention, Dineen reduced his oxycodone consumption from 15 mg/day to 0 in 7 days.

Placebo effects are observed as improvements in the patient's symptoms in the absence of an active drug or medical intervention. This is not only related to the intake of an inert capsule but is bound to all the symbols and interactions associated with the medical interventions. The placebo effect is attributable to the patient's participation in the therapeutic encounter (for Dineen, knowingly taking a placebo capsule to decrease opioid consumption) including “healing rituals,” while the neurobiology of placebos might be involved in promoting the release of endogenous opioids during the evocation phase. This diverse collection of emotions and behaviors include identifiable health care paraphernalia [e.g., pill, capsule, cardamom oil, daily phone calls) that can facilitate emotional and cognitive engagement with clinicians, and promote a positive treatment response (38)]. For Dineen, the “COLP ritual” immersed him into an experience where positive expectations reinforced his will to remove a “risky and dangerous medication.” He engaged in receiving the COLP treatment because he was aware of the opioid's adverse side effects and risks of addiction and also believed that COLP would help him minimize issues associated with the opioids while keeping pain under control. Dineen reported increased pain during the placebo-only evocation period, but continued treatment until the end, highlighting Dineen's commitment to the treatment protocol while maintaining an emotional resilience to tolerate the pain increase. He did not use the rescue pain medication prescribed in case of unbearable pain. Dineen showed that personal motivation, treatment expectations, and communication with clinicians positively impacted his emotional and cognitive factors in pain perception during functional recovery. These elements are relevant in a variety of placebo studies across medical and psychological literature (39).

In this case, we believe the factors that made this intervention successful in reducing opioid consumption were: (1) The patient was highly motivated, (2) he had positive treatment expectations and, (3) he had a close relationship with the treating team (40). In our review of the literature, we identified five randomized clinical trials (RCT) using OLP as the main intervention for pain (Table 2). Two of them were in the area of chronic low-back pain (26, 29). In the first-mentioned study, they included patients with pain duration longer than 12 weeks and were randomized to receive treatment-as-usual (TAU) or OLP for 3 weeks, they found that the placebo treatment was tolerated and reduced pain, disability, and depressive symptoms but didn't affect objective mobility parameters, anxiety or stress.

**Table 2:** Published open label placebo randomized clinical trials for pain management with summary of the results.

**Author**	**Intervention**	**Control**	**Condition**	**Study design**	***N***	**Outcome**	**Results**
Flowers et al. **(author?)** (41)	Open label placebo pills paired with analgesics and opioids	Pain treatment as usual	Post spine surgery patients	RCT	*N* = 51 TAU = 25 COLP = 26	Daily morphine milligram equivalentsPain	- Opioid intake: Patients in the COLP group consumed ~30% less daily morphine milligram equivalents compared with patients in the treatment as usual group - Pain No statistically significant difference between groups
Morales -Quezada et al. (42)	Open label placebo pills paired with opioids	Pain treatment as usual	In -patients with spinal cord injury and polytrauma	RCT	*N* = 20 COLP = 10 TAU = 10	- Daily morphine milligram equivalents - Pain	- Opioid intake: COLP significantly more reduction vs. TAU (*p* = 0.001) - Pain: reduction was significant in COLP group (*p* = 0.005) TAU showed a trend in pain reduction
Kleine -Borgmann et al. (29)	Open label placebo pills	No treatment	Chronic back pain	RCT	*N* = 122 OLP = 63 TAU = 59	- Change in pain intensity. Secondary outcomes: patient -reported functional disability - Spine mobility - Depression - Anxiety - Stress	- Pain intensity: OLP = larger reduction compared to TAU (*p* = 0.001, d 5 20.44) reported - Functional disability: larger reduction in OLP vs. TAU (*p* = 0.020, *d* = 0.44) - Depression scores: larger reduction in OLP vs. TAU (*p* = 0.010, *d* = −0.50) - Mobility parameters: no difference - Anxiety and stress: no difference
Carvalho et al. (26)	Open -label placebo	Treatment as usual (TAU)	Chronic low back pain	RCT	*N* = 83 OLP = 41 TAU = 42	- Total pain score. Back -related dysfunction, assessed on the Roland–Morris Disability	- Pain: OLP greater pain reduction vs. TAU (*P*, 0.001), with moderate to large effect sizes - Disability: OLP more improvement compared to TAU (*p* = 0.001), with a large effect size
Kaptchuk et al. (43)	Open -label placebo	No -treatment controls (NTC)	IBS diagnosed by Rome III criteria	RCT	*N* = 80 NTC = 43 OLP = 37	- IBS Global Improvement Scale (IBS -GIS). Secondary - measures were - IBS Symptom Severity Scale (IBS -SSS) - IBS Adequate Relief (IBS -AR)–IBS Quality of Life (IBS -QoL)	- Global improvement scales (IBS -GIS) OLP = produced significantly higher improvement vs. TAU at midpoint and at endpoint (*p* = 0.001, *p* = 0.002). - Symptom severity (IBS -SSS) OLP greater decrease than TAU at midpoint and at the endpoint of study (*p* = 0.008, *p* = 0.03). - Adequate relief (IBS -AR): greater reduction in OLP vs. TAU - At midpoint and endpoint of the study (*p* = 0.02, *p* = 0.03) - Quality of life (IBS -QoL): Trend favoring OLP

*OLP, Open label placebo; COLP, Conditioning open label placebo; TAU, treatment as usual; NTC, no treatment controls; IBS, irritable bowel syndrome*.

In the second study, they randomized adults reporting persistent low back pain for more than 3 months to take placebo pills OLP or to TAU for 3 weeks, the main outcomes were pain intensity and back-related dysfunction. OLP elicited greater pain reduction and reduced disability compared to TAU. In another study evaluating the effects of OLP in irritable bowel syndrome (IBS), authors found that patients given OLP in the context of a supportive patient-practitioner relationship and a persuasive rationale had clinically meaningful symptom improvement in IBS, and that was significantly better than a no-treatment control group, concluding that placebos administered without deception may be an effective treatment for IBS (43). Two recent studies explored the use of COLP in patients with moderate to severe pain, in the first study, COLP was introduced as a pharmaco-behavioral intervention for opioid reduction in hospitalized patients. This exploratory study included participants suffering from spinal cord injury and polytrauma (42). Results showed that participants in the COLP group significantly reduced total opioid consumption by 66% of morphine equivalents at the end of the intervention period, and the pain was significantly reduced when compared to the TAU group. This was the first study using COLP in a hospital setting, where a short-acting opioid was paired with a placebo capsule as an “honest” intervention for opioid dose reduction. In the study from Flowers et al. (2021), in-patients who underwent spine surgery were included to receive, COLP in the immediate postoperative and after discharge periods to reduce daily opioid use, they found that participants in the COLP group, consumed 30% less daily morphine milligram equivalents compared with patients in the TAU group, with no significant pain differences between groups.

All five studies have shown OLP to be safe and effective in treating pain symptoms by applying principles of conditioning and positive treatment expectations. It is also of relevance that all studies showed the feasibility of using OLP in controlled clinical trials, and that patients (and clinicians) were positive about the use of this approach to manage pain. Moreover, the use of an honest placebo approach overcomes concealment and deception in clinical research, thus decreasing the clinician's liability while providing an opportunity to facilitate provider-patient interactions. This pharmaco-behavioral intervention opens the opportunity for clinical use of placebo as it promotes the patient's active participation in the healing process. The open-label placebo approach requires patient awareness and motivation—Dineen was highly motivated to reduce his opioid intake as well as to achieve full recovery—which may, in turn, lead to “self-regulation” processes associated with homeostatic regulation (e.g., endogenous enkephalins and endorphins release), as the patient is aware of the pharmacological “inert” nature of the placebo. Furthermore, the COLP approach implemented after hospitalization discharge could potentially decrease opioid intake across the community, reduce the risk of addiction, and in turn help with the national opioid crisis.

## Patient Perspective

Dineen reported the COLP paradigm was exciting and easy to follow.

## Conclusion

We present the first case report where conditioning open-label placebo was used as a pharmaco-behavioral intervention to reduce dosage and ultimately terminate opioid treatment after hospital discharge. We showed the feasibility of this intervention within the clinical arena.

## Data Availability Statement

The raw data supporting the conclusions of this article will be made available by the authors, without undue reservation.

## Ethics Statement

The patient approved his data to be used for this report.

## Author Contributions

All authors listed have made a substantial, direct, and intellectual contribution to the work, and approved it for publication.

## Conflict of Interest

The authors declare that the research was conducted in the absence of any commercial or financial relationships that could be construed as a potential conflict of interest.
